# Morphological Changes in the Rat Brain in the Early Post-Resuscitation Period: The Relationship Between Hypoxia-Sensitive Immunohistochemical Markers, Hemodynamic and Acid–Base Status

**DOI:** 10.3390/ijms27146373

**Published:** 2026-07-17

**Authors:** Anastasiya Babkina, Ivan Ryzhkov, Maksim Lyubomudrov, Mikhail Yadgarov, Zoya Tsokolaeva, Ekaterina Boeva, Arkady Golubev

**Affiliations:** Federal Research and Clinical Center of Intensive Care Medicine and Rehabilitology, Moscow 107031, Russia; riamed21@gmail.com (I.R.); mlyubomudrov@fnkcrr.ru (M.L.); myadgarov@fnkcrr.ru (M.Y.); tsokolaevazoya@mail.ru (Z.T.); eboeva@fnkcrr.ru (E.B.); arkadygolubev@mail.ru (A.G.)

**Keywords:** cardiac arrest, brain injury, prolyl hydroxylases, S-100β, acid–base imbalance

## Abstract

The relevance of studying the molecular mechanisms of ischemia–reperfusion brain injury is due to the ongoing need to identify therapeutic targets to improve neurologic outcomes following cardiac arrest. This study aims to identify early morphological and molecular changes in the rat brain in the post-resuscitation period after asphyxial cardiac arrest, as well as to assess the relationship of these changes with hemodynamic and acid–base status. Morphological analysis using immunohistochemistry (IHC) revealed an increase in the intensity of the IHC reaction to S-100β protein in astrocytes of the hippocampal CA1 region in rats 3 hours after resuscitation compared to sham-operated controls (*p* = 0.043). Acid–base disturbances were associated with an increase in the intensity of microglia marker Iba-1 staining in the hippocampal CA4 region (*p* = 0.015). A correlation was found between the intensity of the IHC reaction to PHD1 in cortical neurons and the values of mean arterial pressure (r = −0.699, *p* < 0.001), PaO2/FiO2 ratio (r = −0.580, *p* < 0.005), PaCO2 (r = 0.507, *p* = 0.014). The results of this hypothesis-generating study suggest a potential association between the oxygen-sensing protein PHD1 in cerebral cortical neurons and clinically relevant indicators of post-resuscitation severity after cardiac arrest. Further studies are needed to clarify the role of oxygen-sensing prolyl hydroxylases in the pathogenesis of brain injury in critical illness accompanied by impaired central hemodynamics and acid–base imbalance.

## 1. Introduction

Brain damage resulting from ischemia and reperfusion during cardiac arrest (CA) and subsequent resuscitation is associated with a high risk of death and long-term disability [1]. The importance of research into the mechanisms of post-resuscitation neurological impairment and the relationship between changes in brain function and disorders of its structure were identified by academician V. A. Negovsky in the earliest stages of the development of the science of reanimatology [2,3]. This field still remains relevant since survival after CA remains low: only 3–7% of survivors achieve complete restoration of central nervous system (CNS) function, and 80% of patients with successful return of spontaneous circulation (ROSC) remain comatose or in a persistent vegetative state [4].

The mechanisms of ischemia–reperfusion brain injury are complex and include excitotoxicity, disruption of calcium homeostasis, oxidative stress, release of proapoptotic proteins, microglial activation, mitochondrial dysfunction, and impaired autoregulation of cerebral circulation [5].

In contrast to metabolic changes in the brain, which occur within the first minutes of CA [6], morphological changes in neurons are recorded primarily in the delayed post-resuscitation period, several days after ROSC [7,8]. Depending on the region, structures of the brain, and the subtype of neurons, different susceptibility to ischemia–reperfusion injury is observed [9].

The earliest signs of neuronal damage, dependent on the duration of asphyxia in rats (5 or 7 min), are detected in the thalamic reticular nucleus 4 hours after CA [10]. Chromatin structural changes in cerebral cortex neurons have been described 1 hour after ROSC following 12 min of CA [11]. However, there is no consensus on the timing of irreversible neuronal changes in the post-resuscitation period. Tae et al. revealed Fluoro-Jade B-positive neuronal cells in the hippocampal pyramidal layer only after 2 days [12], whereas Secher et al. detected Fluoro-Jade C-positive neuronal damage in the dentate gyrus hilus and the medial CA1 region of the hippocampus as early as 6 hours after asphyxia CA [13]. Conflicting results may be associated with the different sensitivities of the staining methods used in these studies, as well as different CA duration, 5 and 10 minutes, respectively.

Immunohistochemistry (IHC) significantly expands histological studies by identifying cellular markers of molecular brain changes. An IHC study of TNF-α after 5-min asphyxial CA in rats showed decreased TNF-α staining intensity in CA1 pyramidal neurons after 6 hours, continuing for up to 1 day, and a significant increase 2 days after ROSC. Microglial activation was observed in the CA1 region and dentate gyrus 2–3 days after 12-min asystolic CA [12,14]. On day 4 after 10-min CA, a sharp increase in glial fibrillary acidic protein (GFAP)-positive astrocytes in white matter and an increased number of GFAP-positive astrocytes in gray matter regions were detected [15].

Minor changes detected in experimental morphological studies in the early post-resuscitation period are inconsistent with the severe functional impairments that develop early after CA. The relevance of studying the mechanisms of early ischemia–reperfusion brain injury stems from the need to identify therapeutic targets for mitigation of neurological consequences of CA followed by resuscitation.

Histological analysis is an important component of experimental research, providing the opportunity to identify the morphological substrate of functional disorders. However, histological changes do not always correlate with functional and laboratory data, limiting the applicability of experimental data to clinical practice [9]. Integrating histological analysis with in vivo data is essential for a more complete understanding of brain injury pathogenesis and for identifying therapeutic targets.

Evaluation of blood gases and acid–base balance after ROSC is a crucial routine procedure that largely determines resuscitation and intensive care strategy. Persistent hyperlactatemia for several hours after CA is associated with unfavorable neurological outcome [16]. Current clinical guidelines emphasize the importance of maintaining sufficient partial pressure of arterial oxygen (PaO2) and carbon dioxide (PaCO2), as well as mean arterial pressure (MAP) in the post-resuscitation period to ensure adequate cerebral oxygenation and prevent secondary hypoxic–ischemic brain injury [17,18,19]. Since cerebral blood flow autoregulation is impaired after CA and resuscitation, maintaining optimal blood pressure is essential for cerebral perfusion and, according to Wang et al., is associated with decreased neuronal apoptosis [20].

Given the clinical significance of gas exchange and hemodynamic disturbances in the post-resuscitation period, it is appropriate to examine their relationship with hypoxia-sensitive cellular markers in the CNS in models of CA followed by resuscitation. Of particular interest is the response of oxygen sensor proteins such as hypoxia-inducible factor-1 (HIF-1α) and prolyl hydroxylase domain (PHD), which regulate the response to hypoxia, as well as astro- and microglia, which are involved in maintaining homeostasis in the brain. 

This study aims to identify early morphological and molecular changes in the structures of the rat brain in the post-resuscitation period after asphyxial CA, as well as to assess the relationship of these changes with hemodynamic and acid–base status.

## 2. Results

### 2.1. Parameters of Blood Gases, Acid–Base Balance and Hemodynamics of the Experimental Groups Before Euthanasia

Analysis of blood gases, acid–base balance, and MAP values 3 hours after ROSC revealed abnormalities in both the group of rats with CA and the group of sham-operated animals (Table 1). In CA rats MAP and PaO2 values were significantly lower than in sham-operated rats (*p* = 0.048 for MAP and *p* = 0.035 for PaO2), and acid–base balance disturbances were recorded in the majority of cases (9/10); however, these differences did not remain significant after Benjamini–Hochberg correction (FDR-adjusted *p* = 0.145 for both), although the effect sizes were large (rank-biserial r = 0.51 and 0.56, respectively). Abnormalities in acid–base balance and hyperlactatemia were also detected in sham-operated rats, but to a lesser extent. Respiratory alkalosis (due to hyperventilation) was the most common acid–base balance disturbance in both groups. Decompensated acidosis (both metabolic and respiratory) was observed only in the CA group.

### 2.2. Morphological Changes in the Brain of Experimental Animals

In the cerebral cortex and hippocampus of rats from both groups, shrunken basophilic “dark” neurons, lightly stained “ghost” neurons (characterized by faded cytoplasm, lost nuclear staining, and vague cellular outlines), satellitosis (the clustering of perineuronal glial cells around the neuron), and neuronophagia (the direct cellular invasion of neurons by microglial cells) were detected in each preparation stained with hematoxylin and eosin (Figure 1). Pericellular edema of varying severity was noted around neurons and glial cells. Perivascular edema was observed in all cases studied, regardless of the experimental group. Between-group analysis revealed no differences in the severity of the morphological changes detected in the cortex and hippocampal regions (Table 2).

### 2.3. Intergroup Differences in the IHC Reaction with Antibodies to PHD1, HIF-1α, S-100β, Iba-1 in the Sensorimotor Cortex and CA1, CA4 Regions of the Hippocampus

Immunohistochemical staining of PHD1, HIF-1α in neurons was characterized by varying degrees of staining intensity: from pale single cells to intense staining of each neuron in the studied area. Staining of calcium-binding protein (S-100β) in astroglia and ionized calcium binding adaptor molecule 1 (Iba-1) in microglia was observed in each preparation with varying intensity. Numerous minute S-100β-positive granules, which were probably within the astrocytic processes, were dispersed between neuronal and glial cell bodies. Comparative analysis of the number and intensity of stained cells (reciprocal staining intensity, RSI) in the experimental groups revealed a significant difference in the intensity of the IHC reaction to the S-100β protein in the CA1 region of the hippocampus (*p* = 0.014). This difference remained significant after Benjamini–Hochberg FDR correction within the S-100β RSI regional family (FDR-adjusted *p* = 0.043; rank-biserial r = 0.65). In sham-operated rats, the RSI of S-100β in the CA1 region of the hippocampus was lower than in rats with CA and subsequent resuscitation (Figure 2). The number of PHD1+, HIF-1α+, Iba-1+ cells and their RSI did not differ significantly between the groups of animals (Table 3).

### 2.4. The Relationship Between Hemodynamic Parameters, Acid–Base Balance and Blood Gases with Immunohistochemical Changes in the Brain

In the group of animals with hyperlactatemia (lactate > 2 mmol/L), compared with the group without hyperlactatemia, a higher RSI of PHD1 staining in the cerebral cortex (*p* = 0.033) and a smaller number of HIF-1α-positive cells in the CA4 region of the hippocampus (*p* = 0.021) were found (Table 4). After Benjamini–Hochberg FDR correction, these findings were non-significant (FDR-adjusted *p* = 0.098 for PHD1 RSI in cortex, rank-biserial r = 0.61;FDR-adjusted *p* = 0.062 for HIF-1α-positive cells in CA4, rank-biserial r = 0.71).

The studied IHC markers in the brain were analyzed in groups with different types of acid-base balance disorders: alkalosis (n = 9), acidosis (n = 5), and normal acid–base status (n = 8). It was found that the RSI of Iba-1 staining in the CA4 region of the hippocampus was higher in acidosis than in alkalosis and normal conditions: alkalosis [155.6 (152.4; 157.4)] vs. acidosis [177.1 (154.8; 179.7)] vs. normal [ 151.6 (143.1; 152.9)], *p* = 0.005 (Figure 3). This difference remained significant after FDR correction (FDR-adjusted *p* = 0.015; epsilon-squared = 0.44).

A correlation was revealed between PHD1 RSI in cerebral cortical neurons and MAP (r = −0.699, *p* < 0.001), PaO2/FiO2 ratio (r = −0.580, *p* < 0.005), and PaCO2 (r = 0.507, *p* = 0.014) (Figure 4 and Figure 5). These correlations remained significant after Benjamini–Hochberg FDR correction (all FDR-adjusted *p* values ≤ 0.014).

## 3. Discussion

This study demonstrated an increase in the intensity of the IHC reaction with antibodies to S-100β in the CA1 region of the rat hippocampus in the early post-resuscitation period in rats after CA. No intergroup differences in hypoxia-sensitive markers in the cortex and hippocampus were revealed. The different severity of the animals’ conditions after the experimental procedures, regardless of the experimental group, created the prerequisites for the analysis of the studied molecular markers, taking into account the disturbances in the acid–base balance, blood gases and hemodynamics. Deviations in the arterial blood acid–base balance were associated with an increase in the intensity of microglia (Iba-1) staining in the CA4 region of the hippocampus. Increased PHD1 staining intensity in cortical neurons was associated with decreased MAP and PaO2/FiO2 ratio, and with increased PaCO2.

Depending on the brain region, different reactions of molecular markers to disturbances in hemodynamics, arterial blood gases and acid-base balance were noted. The response of cortical neurons was associated with the oxygen sensor PHD1, whereas in the hippocampus, changes were associated with microglial and astroglial reactions. The hippocampus, striatum, and neocortex are among the most vulnerable brain regions. According to several studies, the neocortex is less sensitive to hypoxia/reperfusion than the hippocampal CA1 region [21]. Regional differences in partial pressure of brain tissue oxygen (PbtO2) should also be taken into account. Experimental data show that within 5 minutes of the onset of acute hypoxia, the partial pressure of oxygen decreased to 8 mmHg at a depth of 100 μm below the surface of the hippocampal slice, and to 45 mmHg at the surface [22]. Manole et al. reported cortical hypoxia despite thalamic hyperoxia in immature rats after 9- and 12-min asphyxial CA, with PbtO2 depending on inspired O2 fraction [23]. Consequently, the different nature of IHC changes in the cortex and hippocampus may reflect both different sensitivity of brain structures to hypoxia and regional features of PbtO2.

In our study, the response to cerebral ischemia–reperfusion was characterized by an increase in the intensity of S-100β staining in the astroglia of the CA1 region of the hippocampus. The remaining IHC markers, according to our study, did not respond to CA followed by resuscitation, but were associated with disturbances in the acid–base balance, blood gases and hemodynamics. The absence of differences between the experimental groups for PHD1, HIF-1α, and Iba-1 may be due to the development of acid–base balance disturbances and hyperlactatemia in both animals with CA and sham-operated animals, which emphasizes the role of individual characteristics of animals in responding to experimental procedures. The reasons for the lack of intergroup differences may be the peculiarities of the short-term CA model (2 min no—flow time), as well as early stages of the post-resuscitation period (3 h).

S-100β is a calcium-binding protein that is primarily produced by astrocytes, oligodendrocytes, and Schwann cells in the CNS and is considered one of the earliest biomarkers of brain injury in CA [24]. Its intracellular functions include signal transmission through regulation of protein phosphorylation, enzyme activity, calcium homeostasis, and astroglia morphology [25]. S-100β may exert neuroprotective and neurotrophic effects, whereas its overexpression may be toxic and contribute to cell death [26]. Jaw et al. showed that reactive astrocytosis and elevated levels of the S-100β subunit may be involved in recovery processes after hypoxic–ischemic brain injury [27].

Increased S-100β protein staining intensity in the hypoxia-sensitive CA1 region may indicate increased expression of this protein in response to cerebral ischemia–reperfusion, consistent with the concept of S-100β as an early clinically relevant marker after CA [26,28,29]. Liu et al. showed that increased serum S-100β content and increased expression of this protein in the hippocampal CA1 region were observed as early as 2 h after ROSC, which is supported by our results [30]. In view of the available literature data on the dual role of increased S-100β expression, it is impossible to draw a definitive conclusion about whether the detected change is a sign of damage or an adaptive response.

The identified associations of the oxygen sensor PHD1 with MAP, PaO2/FiO2 ratio, and PaCO2 are of particular interest due to the clinical significance of these parameters. Studies examining the relationship between metabolic changes in arterial blood and the brain are limited. It has been shown that glucose metabolism in the brain decreases proportionally to the increase in circulating lactate [31]. An experimental study in which animals did not experience hypoxemia or hypotension in the early period after resuscitation showed that PaO2 had a significant relationship with PbtO2, but MAP and etCO2 did not [19]. PaCO2 is an integral parameter of pulmonary gas exchange (hypercapnia is usually accompanied by hypoxemia) and acid–base balance, particularly by significantly affecting cerebrospinal fluid pH and cerebral vascular tone. Therefore, the moderate positive correlation we found between RSI of PHD1 in the brain and PaCO2 may be due to the influence of hypo- and hypercapnia on cerebral perfusion and oxygenation.

Studies examining the relationship between acid–base balance disorders and morphological changes in the brain are rare. Lo et al., using a model of diabetic ketoacidosis in young rats, found reactive gliosis and microglia activation in the hippocampus [32], which is consistent with our results showing an increase in the intensity of microglia staining in the CA4 region of the hippocampus in rats with acid–base balance disorders (acidosis, alkalosis).

Hypoxia sensor proteins play a key role in responding to ischemia/hypoxia and protecting cells from hypoxic damage. Prolyl hydroxylases (PHD1–3) are the main oxygen sensors that maintain low levels of HIF-1α protein under normoxia and, due to decreased activity, rapidly stabilize HIF-1α under hypoxia, facilitating cellular adaptation [33].

The general concept of suppression of PHD function in hypoxia is contradicted by data from a number of studies. Stiehl et al. showed that PHD2 and PHD3 retain HIF-1α degrading activity even under severe hypoxia in cell culture [34]. A time-dependent increase in PHD3, PHD2, and PHD1 expression during hypoxia was detected in nucleus pulposus cells of the intervertebral disc [35]. In glioma cell cultures, oxygen deficiency limited PHD enzymatic activity but stimulated transcription, leading to the greatest PHD activity during reoxygenation [36].

IHC staining intensity does not directly reflect enzyme activity. Based on published data showing increased PHD expression under hypoxic conditions, the observed change may be interpreted as reflecting increased PHD protein abundance in neurons. However, since IHC staining intensity is not linearly related to antigen concentration, this interpretation remains tentative and warrants confirmation by orthogonal quantitative assays.

Since current knowledge of the PHD-dependent hydroxylation reaction and its characteristics depending on oxygen availability is based primarily on data obtained *in vitro* biochemical studies, the interpretation of *in vivo* results remains difficult. Few *in vivo* studies have shown the induction of PHD in hypoxic mice [34]. In a model of ischemic brain injury after right carotid artery occlusion, PHD3 protein and HIF-1α significant increased at 24 h and decreased at 72 h after occlusion [37]. Gerber et al., using an anoxia–reoxygenation model in *Carassius carassius*, showed that PHD expression is upregulated to limit energy-consuming HIF activation during prolonged anoxia and prepare for reoxygenation. Protein levels of all PHD forms in the brain were maintained under anoxia and increased upon reoxygenation [38].

According to *in vivo* and *in vitro* studies, stabilization and accumulation of HIF are adaptive processes that promote cell survival under hypoxic conditions [39]. Experimental studies have demonstrated an increase in HIF-1α protein in the brain after cerebral ischemia [40,41], although its role in limiting or promoting ischemic injury remains controversial and may depend on CNS cell type [42]. HIF-1α has been shown not only to enhance glycolysis and lactate release from cells, but also to increase lactate uptake for gluconeogenesis, which accelerates lactate elimination and reduces the level of lactic acidosis during hypoxia [43]. Wu et al. in an experimental study demonstrated the effectiveness of PHD inhibitors (PHDi) in correcting lactatemia in hemorrhagic shock, associated with the stabilization of HIF, which in turn activates lactate recirculation through gluconeogenesis [44].The association between HIF-1α and hyperlactatemia in our study did not remain significant following Benjamini–Hochberg FDR correction. However, given the limited sample size, a true biological effect cannot be excluded and requires confirmatory research.

The paradoxical results of studies on the consequences of HIF activation can be explained by the complex interaction of responses to hypoxia, the specificity of expression patterns and activity of various HIF and PHD isoforms for neuronal cells of different types [45].

Our findings warrant further research to determine whether the increase in PHD1 response to hypoxemia and decreased MAP is an adaptive response or an initial step in the pathogenesis of brain injury. This field of study is very important, given the numerous studies demonstrating the neuroprotective effect of PHD inhibitors [45,46,47], including PHD1 inhibitors, the effects of which contributed to the regulation of cortical neuronal death caused by oxidative stress [48]. Although the therapeutic potential of PHD inhibitors has been demonstrated in various experimental models, studies in CA followed by resuscitation are lacking. Therefore, the results of the present study and literature data support further investigation of PHD inhibitors in the model of CA followed by resuscitation are lacking. Therefore, the results of the present study and literature data substantiate the advisability of further research on PHD as a therapeutic target in this critical illness model.

The limitations of our study are largely due to the fact that it is exploratory. The analysis we conducted was not foreseen at the experimental design stage. The study’s main limitations include a small sample size, the absence of a group of intact animals, the evaluation of parameters at only one time point, and the use of IHC antibodies to a single PHD isoform. Orthogonal methods were not used to validate the IHC findings. Therefore, changes in staining intensity should be interpreted as indicators of altered immunoreactivity rather than as direct measures of protein abundance or enzymatic activity. In addition, the number of exploratory comparisons was relatively large compared with the sample size. We therefore added FDR-adjusted analyses and effect-size estimates for the main findings, but subgroup results should still be interpreted as hypothesis-generating and require confirmation in independent experiments with a prespecified analysis plan.

Despite these limitations, our findings are scientifically novel and open new prospects for investigating the cerebral PHD response to hypoxic–reperfusion injury during CA followed by resuscitation.

## 4. Materials and Methods

**Experimental material.** This study analyzed histological material from an original experimental trial of the therapeutic potential of argon–oxygen mixture inhalation in the early post-resuscitation period using a model of asphyxial CA [49]. This analysis is additional and was not pre-specified at the experimental design stage.

The original study was conducted on male Wistar rats weighing 200–250 g, N = 43. The animal study was conducted in accordance with accepted national and international bioethical standards [50]. The study protocol was preliminarily approved by the local ethics committee of the Federal Research and Clinical Center of Intensive Care Medicine and Rehabilitology of the Ministry of Education and Science of the Russian Federation (protocol 1/23/4, 5 April 2023). No new animal procedures were performed for the present study. In the original in vivo study, animals were randomized into three groups using random number generation: sham-operated animals (n = 13), animals with asphyxial cardiac arrest (CA) (n = 13), and animals with asphyxial CA treated with an argon–oxygen gas mixture (n = 18). Animals from the latter group were not included in the present post hoc analysis. In the asphyxial CA group, two animals were excluded from the study due to anesthesia-related complications. All remaining animals in this group achieved return of spontaneous circulation (ROSC) after resuscitation, and one animal in the same group died 2 h after ROSC.

All histological material available and preserved in the archive from the original study was included in this analysis, without any prior selection. The present study included data and histological material from animals in the sham-operated group (n = 13) and the group with CA and subsequent resuscitation (n = 10).

All archival tissue blocks were assigned numeric codes by a laboratory research as-sistant not involved in the microscopic analysis. IHC-staining was performed in batches comprising slides from different groups. The investigators performing IHC-staining (Ts.Z.), and subsequent microscopy/morphometry (L.M.) were blinded to the experimental groups of the animals.

**Anesthesia and preparation.** Combined anesthesia was used: tiletamine/zolazepam (Zoletil 100; Virbac, Carros, France) 20 mg/kg + xylazine (Xylanit; NITA-FARM LLC, Saratov, Russia) 5 mg/kg intraperitoneally. To measure blood pressure and obtain arterial blood samples, the left carotid artery was catheterized with a PE-50 polyethylene catheter (OD 0.95 mm, ID 0.58 mm; SciCat, Moscow, Russia).

For drug administration, the right jugular vein was catheterized. Catheters were flushed with unfractionated heparin solution (5 U/mL, 0.1–0.2 mL) as needed. Tracheal intubation was performed with a 16G venous catheter. The animal was connected to a SAR-1000 ventilator (CWE Inc., Ardmore, PA, USA). Rocuronium bromide was administered intravenously at a dose of 1.4 mg/kg body weight. Mechanical ventilation was performed in the CMV/VC mode (FiO_2_ 0.21, f 60/min, I : E 1 : 2, Vt according to the nomogram for rats). The rat was fixed in a supine position on the heated platform of the MouseMonitor S (INDUS Instruments, Webster, TX, USA). The period of stabilization of the animal’s condition before the start of measurements was 15 min.

**Blood pressure measurement.** The arterial catheter was connected to a Deltran transducer using a tee and an infusion line DPT-100 (Utah Medical Products, Inc., Midvale, UT, USA). The analog pressure signal from the transducer and the BP-100 device (CWE Inc., Ardmore, PA, USA) was transmitted to a PowerLab16/35 device (ADInstruments, Sydney, NSW, Australia), connected to a PC. The digitized blood pressure (BP) signal was analyzed using LabChart software Pro 8 (ADInstruments, Pty Ltd., Bella Vista, NSW, Australia). The MAP was calculated from the BP curve over the measurement period (5 min).

**Arterial blood gas analysis and acid–base status.** An arterial blood sample (0.2 mL) was collected from an arterial catheter into a heparinized syringe (1.0 mL). Arterial blood gas and acid–base balance analysis (pH, PaCO2, PaO2, BE, HCO3, SaO2, lactate) was performed using CG4+ reagent cartridges for the i-STAT 1 analyzer (Abbott Point of Care Inc., Princeton, NJ, USA).

**Experimental model of CA and resuscitation.** A previously described experimental model of asphyxial CA in rats was used [51]. After repeated administration of a muscle relaxant (rocuronium bromide 1.4 mg/kg), mechanical ventilation was discontinued. Electrocardiogram (ECG) and blood pressure were monitored to determine the moment of CA. A decrease in mean arterial pressure below 20 mmHg in combination with extreme bradycardia (or other pathological rhythm) was the criterion for the onset of CA. After 2 min (no-flow time) resuscitation measures were performed: mechanical ventilation (FiO_2_ 100%), chest compressions, and intravenous administration of adrenaline 0.005 mg/kg. Simultaneously, ECG and blood pressure monitoring were continued. Upon ROSC, mechanical ventilation at 100% O_2_, blood pressure and ECG monitoring, and infusion of 0.9% NaCl solution at a rate of 10 mL/kg/h were continued. Five minutes after resuscitation, arterial blood gas analysis and acid–base status were assessed, based on the results of which the mechanical ventilation parameters were adjusted. In case of severe metabolic acidosis (pH 7.1, BE ≤ −10 mmol/L), 5% NaHCO_3_ solution was infused at a dose of 1 mmol/kg. Then FiO_2_ in the respiratory mixture was reduced to 30% and mechanical ventilation and vital sings monitoring were continued for 2 h. In the sham-operated group, the same procedures and measurements were performed as in the CA group, except for CA and resuscitation (including without the administration of adrenaline and bicarbonate, but with infusion, anesthesia and muscle relaxants according to the study plan). After 2 h of the post-resuscitation period, the animals were transferred to spontaneous breathing and observation continued for another 1 h. Blood pressure, Arterial blood gases and acid–base status were assessed at the end of this period (3 h after ROSC). Then the animals were euthanized by intravenous administration of 4% KCl solution under general anesthesia with confirmation of asystole on the ECG.

**Histological examination.** Within 15 min after euthanasia, a complete autopsy of the experimental animals was performed. Organs were fixed in 10% buffered formalin (BioVitrum LLC, St. Petersburg, Russia) for 48 h, with a volume of fixative fluid at least 10 times greater than the volume of the specimen. After standard processing, the specimen was embedded in paraffin blocks, and 5 μm-thick frontal sections were cut at the level of the sensorimotor cortex using a rotary microtome. The sections were stained with hematoxylin and eosin. Histological and morphometric analysis of hematoxylin and eosin-stained sections was performed on digital images using an Eclipse Ni-U microscope equipped with a digital camera and NIS-Elements BR software (Nikon Corporation, Tokyo, Japan). In total, 10 images per rat were obtained at 400× magnification: 6 images of the sensorimotor cortex (3 images from each hemisphere) and 4 images were taken at the CA1 and CA4 region of the hippocampus (1 image from each hemisphere).

In each digital image dark neurons, lightly stained ghost neurons, satellitosis, and neuronophagia were counted. The average values for the number of morphologically altered neurons across 6 images were calculated for the sensorimotor cortex, and the total values for 2 images were calculated for the CA1 and CA4 regions of the hippocampus. Perivascular and pericellular edema was assessed semiquantitatively from 0 to 2 points, where 0 means no edema, 1 means moderate edema, and 2 means severe edema. The total score for “pericellular edema” was calculated for each examined brain region per specimen.

**Immunohistochemical study.** For immunohistochemistry (IHC) study, sections were deparaffinized in xylene and sequentially dehydrated in alcohols. Antigen retrieval at high temperatures was performed in citrate buffer with pH 6 (Target Retrieval Solution, DAKO, Glostrup, Denmark). Sections were cooled, washed in three changes of distilled water, and incubated in 3% hydrogen peroxide to inhibit endogenous peroxidase activity. Sections were then washed with phosphate buffer (3 × 5 min) (PBS IHC Wash Buffer + Tween, Cell Marque, Rocklin, CA, USA). To prevent nonspecific binding of primary or secondary antibodies to tissue proteins, blocking serum (Protein Block Serum-free, Abcam, Cambridge, UK) was used for 15 min. Sections were then incubated for 1 h at 37 °C with primary antibodies against HIF-1α (ab114977; Abcam, Cambridge, UK; 1:100), PHD1/prolyl hydroxylase (ab113077; Abcam, Cambridge, UK; 1:300), Iba-1 (ab178846; Abcam, Cambridge, UK; 1:300), S-100β [EP1576Y] (ab52642; Abcam, Cambridge, UK; 1:100) diluted in Antibody Diluent (ab64211 Abcam, Cambridge, UK). Sections were washed with PBS (2 × 5 min). Goat Anti-Rabbit IgG H&L (ab 214880 Abcam, Cambridge, UK) was used as the secondary antibody. The sections were then incubated with 3,3′-diaminobenzidine (DAB) substrate using a DAB Substrate Kit (ab64238; Abcam, Cambridge, UK) according to the manufacturer’s instructions. The staining was monitored under a light microscope until desired staining intensity was achieved (∼2–3 min). The reaction was stopped by washing in PBS four times for 1 min each. After the final rinse, the sections were counterstained with hematoxylin and coverslipped.

Whole-slide digital images of the IHC-stained brain sections were acquired using an Aperio ScanScope CS slide scanner (Aperio/Leica Biosystems, Vista, CA, USA) at 20× objective magnification under standardized acquisition conditions to minimize variability in image back-ground. From the digitized whole-brain images, three non-overlapping areas within the sensorimotor cortex and two hippocampal areas (CA1 and CA4) free of artifacts were randomly selected within one hemisphere. Representative images were captured at the highest available magnification using the Extract Region tool in Aperio ImageScope soft-ware (Version 12.4.6.5003; Leica Biosystems, Vista, CA, USA).

Quantifications (the cell count and mean intensity of stained cells in each digital image) were performed using NIS-Elements BR (Basic Research) software version 5.20 (Ni-kon Corporation, Tokyo, Japan). Color thresholding was performed using the Thresholding tool in the HSI (Hue, Saturation, Intensity) color space to identify positively stained regions. Thresh-old values were determined separately for each immunohistochemical marker using rep-resentative sections and were subsequently applied uniformly to all digital images of sec-tions IHC-stained for that marker.

Regions of interest (ROIs) were defined based on DAB-positive staining in brain cells (neurons, microglia, and astroglia). Positive IHC-staining outside ROI (e.g., artifacts, blood vessels) was manually corrected. The mean intensity of the selected objects was determined automatically in NIS-Elements software for each acquired digital image, with each image corresponding to one field of view. For each animal, average values for the number of stained cells and their Mean Intensity were calculated in the sensorimotor cortex and the CA1 and CA4 hippocampal regions.

Pixel intensity values in NIS-Elements range from 0 to 255, where 0 is the darkest shade and 255 is the lightest shade. To simplify the perception of the results in tables and graphs, instead of the Mean Intensity values, the DAB reciprocal staining intensity (RSI) where RSI = 255—mean intensity, was used [52].

**Parameters.** To assess the relationship of hemodynamic parameters, arterial blood gases and acid–base status with morphological and immunohistochemical changes in the brain, the following parameters were measured before euthanasia (3 h of the post-resuscitation period): MAP, pH, PaCO2, PaO2, base excess/deficiency (BE), bicarbonate concentration (HCO3-), arterial blood saturation (SaO2) and lactate. Based on these parameters, the type of acid–base balance disorder and PaO2/FiO2 ratio were determined.

To assess the response of the studied IHC markers to acid–base balance disturbances noted before euthanasia, animals from both experimental groups were divided into groups based on the following criteria: type of acid–base balance disturbance (acidosis; alkalosis; normal) and the presence of hyperlactatemia (lactate > 2 mmol/L; lactate < 2 mmol/L). A comparative analysis of the number and RSI of IHC-stained cells between the isolated groups of animals was then conducted. Individual animal-level data, including hemodynamic parameters, arterial blood gases, acid–base status, lactate levels, and quantitative histological and immunohistochemical measurements, are available in Supplementary Table S1. Additional representative rat brain microphotographs, together with the corresponding binary masks used for quantitative histological image analysis, are openly available in the Mendeley Data repository [53].

**Statistical analysis.** Statistical analysis was performed using IBM SPSS Statistics 29.0 (IBM Corp., Armonk, NY, USA) and the Python 3.13 programming language.

The single animal was considered the statistical unit for all analyses. Quantitative indicators were described using the median and interquartile range (IQR)—Me (Q1;Q3). Qualitative indicators were presented as absolute values and proportions, n (%). Missing values were not imputed; analysis was performed using available observations for each specific indicator.

The Mann-Whitney U test was used to compare two independent groups based on quantitative and ordinal variables. Fisher’s exact test was used to compare qualitative variables; when analyzing contingency tables larger than 2 × 2, the Fisher-Freeman-Halton test was used. When comparing more than two independent groups, the Kruskal-Wallis test was used. For two-group comparisons, the rank-biserial correlation was used as a nonparametric effect size. For the three-group analysis, epsilon-squared was used as the effect size.

To assess the relationship between immunohistochemical parameters and hemodynamic parameters, blood gas composition, and arterial blood acid–base balance recorded before euthanasia, a correlation analysis was performed using Spearman’s rank correlation coefficient (ρ). All statistical tests were two-sided. Differences were considered statistically significant at *p* < 0.05. Multiplicity was addressed using the Benjamini-Hochberg false-discovery-rate correction within biologically related local families of comparisons: CA versus sham comparisons, lactate subgroup comparisons, and acid–base status subgroup comparisons, each assessed across the three brain regions separately for each parameter.

## 5. Conclusions

This study suggests that the early response to cerebral ischemia–reperfusion in an asphyxial cardiac arrest model is characterized by increased S-100β immunohistochemical staining intensity in astroglia of the hippocampal CA1 region. A two-minute asphyxial cardiac arrest did not produce detectable changes in PHD1, HIF-1α, or Iba-1 immunoreactivity in either the cerebral cortex or hippocampus. However, PHD1 immunostaining in the cerebral cortex and Iba-1 immunostaining in the CA4 region of the hippocampus appear to be associated with systemic disturbances, including alterations in acid–base status, gas exchange, and hemodynamic parameters. Specifically, increased PHD1 staining intensity in cortical neurons was associated with lower MAP and PaO2/FiO2 ratio, as well as with higher PaCO2.

The results of this hypothesis-generating study point to a possible relationship between the oxygen-sensing protein PHD1 in cerebral cortical neurons and clinically relevant indicators of post-resuscitation severity after cardiac arrest. Further studies are needed to clarify the role of oxygen-sensing prolyl hydroxylases in the pathogenesis of brain injury in critical illness accompanied by impaired central hemodynamics and acid–base imbalance.

## Figures and Tables

**Figure 1:**
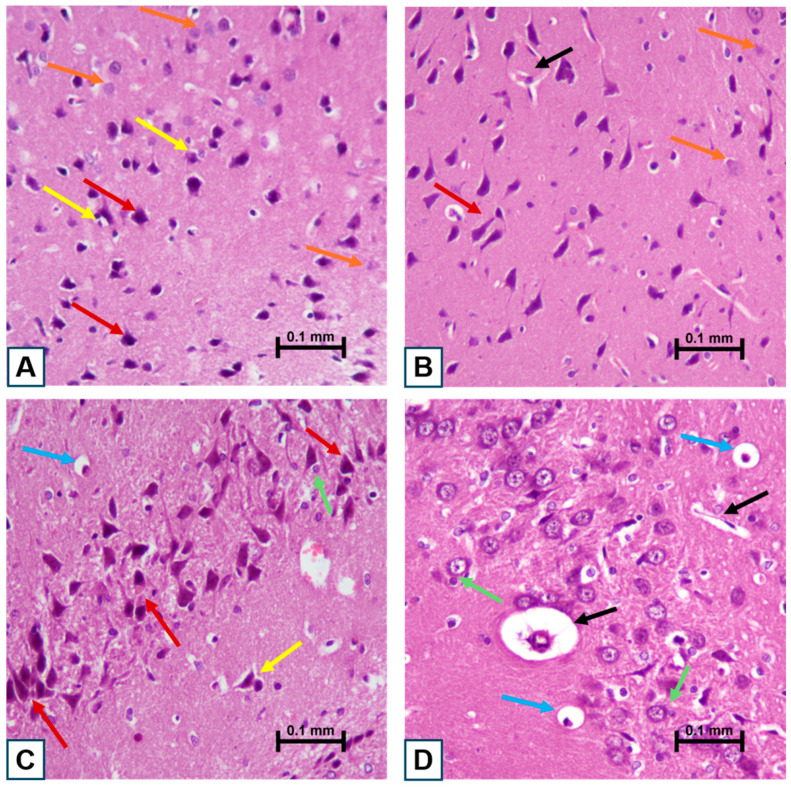
Morphological changes in the cortex (**A**,**B**) and hippocampus (**C**,**D**) of the rat brain of the experimental groups. Hematoxylin and eosin staining. Magnification, ×400. Neuronophagia (shown by yellow arrows, (**A**,**C**)), satellitosis (shown by green arrows, (**C**,**D**)), dark neurons (shown by red arrows, (**A**–**C**)), lightly stained ghost neurons (shown by orange arrows, (**A**,**B**)), perivascular edema (shown by black arrows, (**B**,**D**)), pericellular edema (shown by blue arrows, (**C**,**D**)).

**Figure 2:**
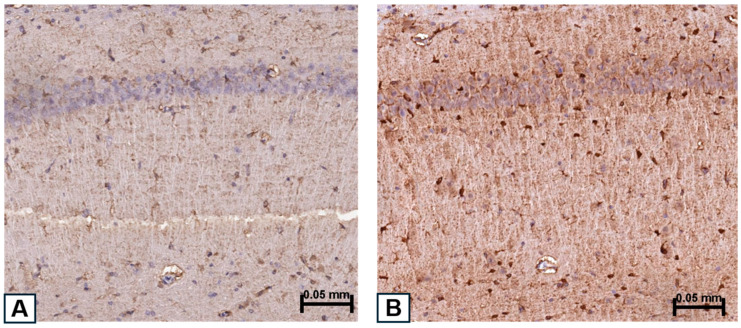
Representative image of IHC staining of S-100β in the CA1 region of the hippocampus. Magnification, ×200. (**A**) Sham-operated rat brain section. (**B**) CA rat brain section.

**Figure 3:**
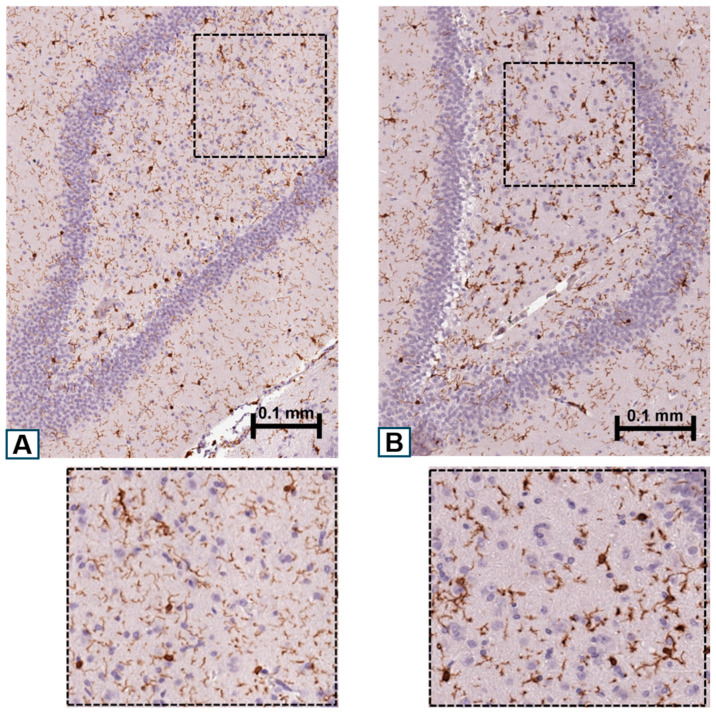
Representative image of IHC staining of Iba-1 in the CA4 region of the rat hippocampus without acid-base disorders (**A**) and with acidosis (**B**).

**Figure 4:**
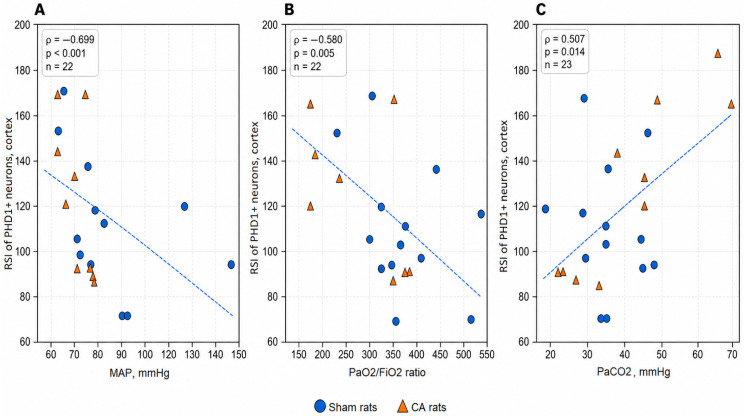
Correlation between the RSI of PHD1 in cortical neurons and MAP (**A**), PaO2/FiO2 ratio (**B**) and PaCO2 (**C**).

**Figure 5:**
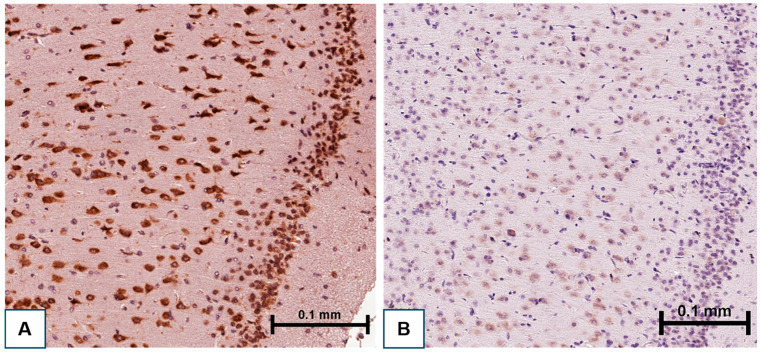
Representative image of IHC-staining for PHD1 in the rat cerebral cortex. Magnification ×200. (**A**) Cerebral cortex section from a rat with MAP of 57 mmHg, PaO2/FiO2 ratio of 161; (**B**) Cerebral cortex section from a rat with MAP of 145.5 mmHg, PaO2/FiO2 ratio of 352.

**Table 1:** MAP, parameters of gases and acid-base balance of arterial blood in sham-operated and CA rats.

Parameters	Sham-Operated Rats, n = 13	CA Rats, n = 10	*p*-Value
MAP	77.9 (71.4; 93.9)	70.7 (63.2; 76.6)	**0.048**
pH	7.426 (7.394; 7.483)	7.369 (7.263; 7.493)	0.352
PaCO2	35.0 (29.5; 44.6)	41.4 (26.1; 52.7)	0.476
PaO2	78.0 (70.0; 90.5)	53.0 (41.0; 77.5)	**0.035**
BE	0.0 (−3.0; 2.0)	−2.5 (−6.3; 0.3)	0.171
HCO_3_	23.6 (21.4; 26.4)	21.6 (18.7; 25.6)	0.368
SaO_2_	95.0 (93.5; 97.5)	85.0 (64.5; 96.5)	0.060
Lactate	1.04 (0.85; 2.32)	0.97 (0.71; 2.53)	0.710
Type of acid–base status disorder, n (%)			**0.031**
- respiratory acidosis	0 (0.0%)	2 (20.0%)	
- respiratory alkalosis	4 (30.8%)	5 (50.0%)	
- metabolic acidosis	0 (0.0%)	2 (20.0%)	
- mixed acidosis	1 (7.7%)	0 (0.0%)	
- metabolic acidosis compensated by respiratory alkalosis	1 (7.7%)	0 (0.0%)	
- normal	7 (53.8%)	1 (10.0%)	
PaO2/FiO2 index	362.0 (323.5; 428.5)	252.0 (195.0; 369.0)	0.066
Lactate > 2 mmol/L	4 (30.8%)	2 (20.0%)	0.660

The statistically significant results are highlighted in bold. Quantitative values are presented as median (interquartile range), and categorical variables as n (%). Mann–Whitney U test was used for quantitative variables, Fisher exact test for binary categorical variables, and Fisher-Freeman-Halton test for the acid-base status distribution. Multiplicity handling is described in the Statistical analysis section.

**Table 2:** Morphological changes in the brain of sham-operated and CA rats.

Parameters	Cortex Sham	Cortex CA	*p*-Value	CA1 Sham	CA1 CA	*p*-Value	CA4 Sham	CA4 CA	*p*-Value
Dark neurons	10.0 (5.9; 17.9)	6.5 (4.8; 15.5)	0.420	3.0 (0.0; 9.5)	4.0 (0.5; 8.5)	0.886	13.0 (2.0; 25.5)	10.0 (1.5; 30.0)	0.947
Satellitosis	7.5 (5.6; 8.4)	7.1 (5.9; 8.1)	0.709	9.0 (5.8; 11.5)	8.0 (8.0; 13.0)	0.660	11.0 (7.0; 13.0)	12.0 (9.5; 14.5)	0.331
Neuronophagy	0.2 (0.2; 0.6)	0.2 (0.2; 0.9)	0.631	0.0 (0.0; 1.0)	0.0 (0.0; 0.5)	0.687	0.0 (0.0; 0.0)	0.0 (0.0; 1.0)	0.495
Pericellular edema	4.0 (3.0; 5.5)	4.5 (3.8; 8.5)	0.360	0.5 (0.0; 1.0)	1.0 (0.5; 1.5)	0.268	3.0 (2.0; 4.0)	2.0 (1.5; 3.0)	0.201
Lightly stained ghost neurons	10.8 (8.5; 12.7)	7.4 (6.1; 10.8)	0.094	0.0 (0.0; 0.0)	0.0 (0.0;0.0)	1.000	0.0 (0.0; 1.5)	0.0 (0.0; 0.0)	0.387

Values are presented as median (interquartile range). Morphological counts are reported as average counts per field for the cortex and as total counts across two fields for CA1 and CA4. Mann–Whitney U test was used for between-group comparisons.

**Table 3:** Comparative analysis of the number and RSI of IHC-positive cells in the cortex and hippocampal regions of the brain of rats in experimental groups.

Proteins	IHC Parameters	Cortex Sham	Cortex CA	*p*-Value	CA1 Sham	CA1 CA	*p*-Value	CA4 Sham	CA4 CA	*p*-Value
PHD1	IHC-positive cells, average count/field	474.3 (304.8; 524.0)	373.3 (261.8; 498.9)	0.368	86.0 (48.8; 174.0)	92.0 (17.5; 126.0)	0.456	152.0 (142.5; 215.8)	142.5 (114.5; 186.8)	0.468
	RSI	104.5 (92.7; 127.3)	126.1 (89.9; 165.6)	0.515	115.9 (110.3; 137.6)	122.7 (115.3; 142.2)	0.241	104.2 (96.7; 121.5)	121.2 (95.8; 150.1)	0.410
HIF-1α	IHC-positive cells, average count/field	80.0 (15.3; 272.5)	127.0 (109.6; 340.8)	0.163	6.5 (0.2; 80.5)	110.0 (0.0; 147.5)	0.196	86.0 (0.2; 141.5)	75.0 (37.5; 121.8)	1.000
	RSI	99.1 (77.5; 143.2)	108.3 (81.0; 153.1)	0.733	116.0 (91.6; 226.0)	152.1 (115.9; 255.0)	0.196	92.9 (84.1; 233.3)	97.5 (86.8; 162.8)	0.869
S-100β	IHC-positive cells, average count/field	105.3 (98.5; 113.5)	104.2 (87.7; 119.0)	1.000	63.0 (50.8; 73.0)	67.0 (60.0; 78.0)	0.284	127.5 (110.8; 157.5)	175.5 (117.0; 221.0)	0.086
	RSI	152.2 (140.6; 165.4)	162.9 (160.7; 183.2)	0.172	93.8 (82.5; 106.3)	109.0 (99.2; 136.0)	**0.014**	150.8 (136.7; 175.3)	164.4 (134.7; 186.7)	0.717
Iba-1	IHC-positive cells, average count/field	43.3 (40.0; 51.5)	45.0 (37.4; 48.5)	0.687	26.5 (22.2; 31.2)	30.0 (24.0; 37.0)	0.522	57.0 (52.0; 70.0)	55.5 (47.8; 64.8)	0.551
	RSI	155.2 (152.5; 157.9)	158.3 (153.3; 179.5)	0.278	168.5 (159.0; 171.5)	165.8 (147.9; 171.8)	0.749	152.8 (151.5; 156.9)	155.2 (152.6; 177.4)	0.373

The statistically significant results are highlighted in bold. Values are presented as median (interquartile range). Mann–Whitney U test was used for between-group comparisons.

**Table 4:** The number and RSI of IHC-positive cells in the cortex and hippocampus of the rat brain in high and low lactate groups.

Proteins	IHC Parameters	Cortex Lact. Low	Cortex Lact. High	*p*-Value	CA1 Lact. Low	CA1 Lact. High	*p*-Value	CA4 Lact. Low	CA4 Lact. High	*p*-Value
PHD1	IHC-positive cells, average count/field	351.0 (284.2; 502.0)	494.5 (334.6; 543.2)	0.278	87.0 (36.5; 126.0)	89.5 (56.5; 150.3)	0.754	155.0 (133.5; 215.5)	144.0 (72.0; 198.0)	0.530
	RSI	96.5 (88.9; 126.1)	144.0 (114.5; 170.2)	**0.033**	115.8 (110.3; 135.4)	133.6 (119.6; 144.4)	0.139	107.1 (95.7; 127.5)	135.3 (106.7; 160.5)	0.100
HIF-1α	IHC-positive cells, average count/field	133.7 (85.2; 358.0)	69.8 (17.8; 124.8)	0.074	58.0 (2.0; 120.5)	0.5 (0.0; 82.8)	0.221	121.0 (45.0; 141.0)	33.0 (0.0; 46.5)	**0.021**
	RSI	95.7 (78.2; 143.6)	125.6 (80.4; 153.1)	0.506	116.9 (94.6; 205.5)	203.5 (133.2; 255.0)	0.113	92.9 (85.8; 118.0)	172.4 (116.8; 255.0)	0.117
S-100β	IHC-positive cells, average count/field	105.3 (98.5; 115.2)	99.2 (83.8; 109.7)	0.551	67.0 (56.5; 74.5)	57.5 (51.5; 65.0)	0.177	141.0 (112.0; 185.0)	119.0 (113.0; 194.0)	0.875
	RSI	163.4 (154.5; 168.7)	146.2 (129.1; 180.5)	0.344	102.3 (92.0; 112.9)	100.4 (85.2; 108.4)	0.754	155.7 (134.5; 178.9)	150.5 (137.6; 179.4)	0.938
Iba-1	IHC-positive cells, average count/field	47.3 (40.3; 51.7)	40.7 (39.5; 42.8)	0.123	28.0 (22.5; 32.5)	26.0 (22.3; 38.0)	0.964	56.0 (48.0; 68.0)	55.0 (53.5; 68.0)	0.814
	RSI	156.0 (152.3; 159.3)	157.5 (154.4; 183.3)	0.248	168.4 (159.4; 171.4)	167.2 (143.4; 172.1)	0.964	153.2 (151.2; 157.1)	155.9 (153.2; 179.7)	0.183

The statistically significant results are highlighted in bold. Values are presented as median (interquartile range). Mann–Whitney U test was used. For lactate subgroup analyses.

## Data Availability

The data presented in this study are available in Supplementary Materials.

## Supplementary Materials

The following supporting information can be downloaded at: https://www.mdpi.com/article/10.3390/ijms27146373/s1.
